# Rape Perpetrated on a Female While under the Influence of Ether

**Published:** 1847-10

**Authors:** 


20. *Rape perpetrated on a Female while uuder the Influence of Ether.*—That which had been suspected as a probable result, on the introduction of the new narcotizing agent, has, according to the *Gazette Medicale*, actually occurred in Paris. A young woman went to a dentist to have a tooth extracted. To avoid the pain, she was persuaded to inhale the vapor of ether. While under its influence she was violated. The dentist has been arrested.—*Med. News*.

